# Beyond different levels: embodiment and the developmental system

**DOI:** 10.3389/fpsyg.2014.00929

**Published:** 2014-08-20

**Authors:** Peter J. Marshall

**Affiliations:** Department of Psychology, Temple UniversityPhiladelphia, PA, USA

**Keywords:** levels of explanation, developmental systems, multilevel analysis, philosophy, developmental psychology

## Abstract

The value of studying a phenomenon at multiple levels of analysis is often emphasized in psychology, but a lack of clarity about the nature of levels and the relations among them remains an impediment to progress. The suggestion here is that an approach combining the tenets of embodiment with the construct of the developmental system provides a way forward. Embodiment opposes the splitting off and elevation of a level of mechanisms that has characterized much of cognitive science. In contrast, a constructivist embodied approach places a level of mechanisms in the context of a formal or systems level of analysis, with developmental process framing the interpenetrating relations between levels. Such an approach stems from a relational worldview that opposes conceptual splits and posits that levels of structure and process comprise an indissociable complementarity. The combination of embodiment and developmental systems within a relational worldview is discussed and elaborated through outlining the integrative approach of relational developmental systems, which has been proposed as a scientific paradigm within which formulations of the interrelations among brain, body, and mind can be advanced.

The value of explanations spanning multiple levels of analysis has become an important emphasis in psychological science, yet a coherent framework for explicating such levels and the relations among them remains elusive. Within the field of cognitive science, one influential attempt to conceptualize different levels of analysis was put forward by the vision scientist David Marr (1982). In line with the computational emphasis that characterized cognitive science at the time he was writing, Marr's account concerned three levels “at which any machine carrying out an information processing task must be understood” (Marr, 1982, p. 25). The first level, which Marr called the *computational* level, concerns the general nature of the problem or task at hand. At the second level of *representation and algorithm*, a sequence of operations and a representational format is specified that would solve the problem specified at the first level. At the third level of *implementation*, the question is how that particular solution could be realized on a machine (i.e., a description of the physical hardware needed). There are similarities between Marr's account and other levels-based proposals from the same era (e.g., Simon, 1969; Dennett, 1971; Wimsatt, 1976), but his model has remained particularly influential. However, on closer examination, two particular issues constrain the utility of this basic framework (see also Marshall, 2013, in press).

First, psychological science has often been characterized by a tendency to emphasize the explanatory priority of one level over another. For example, it could be argued that cognitive science has historically been too concerned with Marr's second level of representation and algorithm, or the level of problem-solving in terms of what symbols are needed for a solution, and the rules under which those symbols can be manipulated. This emphasis can be partly traced to the influence of the idea that cognition consists of formal computational reasoning processes acting on the syntactic, but not the semantic, aspects of symbolic representations (Fodor, 1975). This cognitivist approach was associated with an alignment of cognitive psychology with the emerging discipline of artificial intelligence, which further contributed to the dominance of an information processing view of the mind (Newell et al., 1958). From this perspective, cognitive operations could be seen as manipulations of sub-personal representations to which meaning had been pre-assigned (for a recent critique, see Allen and Bickhard, 2013). It has been argued in various places that the move toward cognitivism, with its associated emphasis on Marr's second level, was fundamentally a wrong turn in that it prevented the emergence of more integrative accounts of mental life (see Bruner, 1990; Thompson, 2007; Rowlands, 2010).

Second, psychology as a discipline has not arrived at a clear formulation of how to conceptualize the relations between levels. Indeed, it could be argued that the lack of a coherent explanatory framework for understanding the relations between different levels is one of the biggest obstacles to progress in the discipline. This problem can be partly traced to an emphasis within cognitive science on the relative autonomy of each of Marr's levels, which in turn stemmed from the proposal that a given task or problem could be solved in a myriad of ways, using different representational systems or forms of physical implementation (Fodor, 1975; Putnam, 1975; Pylyshyn, 1984). While this notion of *multiple realization* appears to avoid the problem of causal reductionism (Miller, 2010), it sidesteps the crucial question of how to conceptualize the relations among levels.

Given the lack of coherence concerning the nature of levels and the relations among them, how are we to move forward? The suggestion here is that a framework that recognizes the interpenetrating nature of the relations between levels, and in which considerations of development play a key role, is a way forward. More specifically, it is argued that a *relational developmental systems* approach (Overton, 2013), in which the interconnections among levels can be articulated within the context of *embodiment*, provides a route toward a truly integrative account.

## Embodiment

Embodied approaches have become increasingly visible in psychology over the past three decades (e.g., Varela et al., 1991; Damasio, 1994; Glenberg, 1997; Clark, 1998; Anderson, 2003; Wheeler, 2005; Thompson, 2007; Barsalou, 2008; Beer, 2008; Overton, 2008; Semin and Smith, 2008; Menary, 2010). Although there are clearly different theoretical and empirical strands of embodied cognition (Wilson, 2002; Kiverstein, 2012), to a greater or lesser extent they all challenge the isolated computational mind of cognitivism, which lacks a brain, a body and a culture (Edelman, 1992).

By locating the brain in the body of an active, agentive organism, embodiment threatens the clear distinctions between perception (input), cognition (information processing) and action (the execution of instructions or output) that underpin the cognitivist account. One key tenet of embodied approaches is that cognition can no longer be packaged into an isolated level of information processing, or Marr's second level of representation and algorithm. As noted by Clark (2000), “our notions of what top-level task needs to be performed, and what kinds of algorithms are adequate to perform it, are deeply informed by reflection of details of bodily implementation, current needs, and action-taking potential” (p. 96). As such, embodiment puts pressure on a tidy separation of levels (or the isolation of any one level), and the need to understand the relational ties among levels moves to the fore.

Embodiment places the organism as an active agent that is tightly interconnected with its environment, with the actions of the individual constantly modifying these interconnections, a process that in turn influences subsequent actions. In one particular theoretical approach to embodiment, this feedback loop is the foundation of a dynamic system in which the boundaries between individual and environment cannot be clearly determined (Stewart et al., 2010). In turn, this proposal brings with it some far-reaching suggestions. Specifically, advocates of what Chemero (2009) terms *radical embodied cognitive science* suggest that the dynamic coupling of organism and environment has two related implications for framing the study of mental life (see also Hutto and Myin, 2012). First, that cognitive processes are distributed across the dynamic system that results from the non-linear coupling of individual and environment. Second, that the formulation of the wider cognitive system as a dynamic system challenges the need to invoke the concept of representation in accounts of mental life (Silberstein and Chemero, 2012). This challenge is partly founded in the work of Gibson (1979), who proposed that preexisting environmental structure largely negates the need for the concept of mental representation as it is usually understood.

In line with these points, empirical work from the radical embodied perspective often draws on dynamical systems theory as a basis for modeling the coupling of an agent's behavior over time with the changing state of the environment. However, it would be misleading and potentially damaging if an embodied approach was equated with one particular flavor of dynamic systems models. Among others, David Witherington has argued that a full understanding of living things entails seeing levels of organization and process as being complementary and indissociable (e.g., Witherington, 2011; Witherington and Heying, 2013). He makes the point that this stipulation pushes against the Gibsonian emphasis that is apparent in certain flavors of dynamic systems theory, for instance that of Thelen and Smith (1994). According to Witherington (in press), embodiment could be productively aligned with an approach more resembling Piagetian constructivism (see also Witherington and Margett, 2011), a sentiment that would be endorsed by those dynamical systems practitioners who see constructivism as being fundamentally consistent with systems approaches (e.g., van Geert, 2011).

## Relational developmental systems

Here I wish to highlight the suggestion that a particular constructivist approach to embodiment, informed by specific lines of systems thinking in developmental science and the philosophy of biology, has a great deal of potential for informing the understanding of different levels of analysis. This approach is termed *relational developmental systems* (RDS), as put forward by Willis Overton and Richard Lerner, who have suggested that it has key implications for understanding the nature of levels and the relations between them (Overton and Lerner, 2012; Overton, 2013). As the term suggests, RDS combines two broader metatheoretical streams: relationism and developmental systems. The worldview of relationism rejects any simple notion of separable causes, and can be contrasted with what Overton (2006) terms a Cartesian worldview that encourages dichotomies, elevates the explanatory value of proximate mechanisms, and precludes integration. Working under the umbrella of relationism allows these constraints to be jettisoned and enables a move toward a more integrative, developmentally oriented account of brain, body, and mind.

At a finer grain of theory, RDS is further informed by the developmental systems approach that emerged from a particular strand of psychobiological research in the 20th century (Lehrman, 1953; Schneirla, 1959; Gottlieb, 1970) and which brings together related viewpoints from developmental and evolutionary biology (Oyama, 1985; Griffiths and Gray, 1994). While this strand consists of various threads with different emphases (Johnston, 2010; Griffiths and Tabery, 2013), at its core are the notion of the developmental system, the necessity of multiple modes of explanation, and the stipulation that no single aspect of the system can be elevated in terms of its causal role (Shea, 2011). In turn, the developmental systems approach has its roots in principles derived by embryologists in the mid-20th century (e.g., Spemann, 1938; Kuo, 1939) who documented how organismic development proceeds through a process of differentiation and integration. This foundational notion went on to influence developmentalists such as Werner (1948) and Piaget (1952) who laid the foundations for a biologically-informed developmental science of life and mind.

Drawing on the construct of the developmental system, RDS embraces several forms of explanation and brings them together in a relational framework. One key emphasis is on the importance of what can be called *pattern explanation*, or what Overton (1991) labeled *competence*. In turn, the notion of competence is similar to Aristotle's notion of the *formal cause*, which is interrelated with, but different from, other types of explanation such as efficient or material causes (Caston, 2006). It is important here to emphasize the necessarily abstract quality of pattern explanation, which transcends the framing of temporally related antecedents and consequences that is usually associated with the notion of causation. As such, pattern explanation refers to the structure or organization of the endogenously active system. This abstraction reflects the view that organization is not something that exists over and above the parts of a system, yet at the same time allowing organization more than a descriptive role. In this sense, the notion of organization as constraint (Thompson, 2007; Deacon, 2012) is helpful. As framed by Witherington (in press):

“the explanatory causality of a system's organization rests in its top-down constraint. Constraint involves a lessening of variability, a narrowing of degrees of freedom, and as such plays a critical role in causal explanation by virtue of establishing limitations for what kinds of bottom-up processes… are available to a given system; thus, the nature of local interactions cannot be fully understood divorced from the organizational whole in which these interactions are embedded” (p. 90).

The necessity of relating multiple modes of explanation is central to the RDS approach, in which pattern explanation provides the meaning context for a different and complementary level of *processes*, or what Overton (1991) labeled *procedures*. In referring to distinct, observable factors having a casual action that precedes a specific effect, processes (or what in Aristotelian terms would be efficient causes) are quite close to everyday notions of causation. However, as discussed by Witherington (2011, in press), this can too easily lead to a diminished role for structure and a denial of the explanatory import of the formal patterns. According to accounts that discount a causal role for pattern explanation, the appearance of structure arises from the operation of complex positive and negative feedback processes, but does not causally influence the subsequent operation of those processes. However, this neglects the fact that complex processes must be organized in some way, and it is this issue that necessitates the formal level of explanation, which becomes the *system* of a systems approach. Simply put, it is a mistake to believe that pattern explanations are rendered unnecessary if enough processes are described. Adopting such a position would present a conundrum that stretches far back in the history of philosophical and scientific thought, which is that every efficient cause or mechanism cannot be caused by another efficient cause or mechanism. In contrast, from a relational viewpoint, form and process can be seen as inextricably linked through the notion of *circular causality* (Witherington, 2011). Any living system acts according to its particular organization, and that organization changes through its activity.

Perhaps the most problematic manifestation of the neglect of pattern explanation comes through a situation in which processes—as properties of parts of a system—are conflated with the properties of the whole system. In their critique of cognitive neuroscience, Bennett and Hacker (2003) termed this the *mereological fallacy*, such that an accumulation of neural mechanisms cannot stand in as a full explanation of the properties of the individual person. Related instances of conflating subpersonal processes with personal-level properties of the individual are a widespread problem in many areas of contemporary psychology, including developmental science (for discussion of one example, see Rakoczy, 2012). Avoiding these pitfalls requires the understanding that processes at the procedural level must be organized in some way, and that in and of themselves, processes or mechanisms have no context. It is this issue that brings the focus to competence or formal explanation as a different level of analysis, with the stipulation that this level provides a functional context for a different, complementary level of processes.

Given the above, we can move toward seeing the importance of a dynamic pattern that entails an indissociable relation between organization and activity. To use the terminology of Overton (1991), if the level of procedures is understood as the active processes through which competence comes into being, while simultaneously the competence level serves as a context for organizing the procedural level, we can begin to understand how the two levels operate in a complementary fashion. This allows arrival at a relational frame in which the interleaving of pattern explanation and the understanding of specific processes is appreciated as being fundamental to the scientific enterprise (Overton, in press).

A relational perspective on the different levels of structure and mechanism also brings considerations of change and transformation to the fore (Overton, 1991), because the reciprocal relations between the levels must be seen in the context of the developmental process itself. From the viewpoint of RDS, the dynamic tension between competence (pattern explanation or system) and procedures (specific processes) becomes the basis of an inherently developmental, constructivist perspective. As circular causation, the developmental process recognizes both the emergence of form through process along with the constraining (downward) influence of form on process (Witherington, 2011, 2014).

Through an awareness of circular causality, we can begin to understand how the relational and inherently developmental ties between levels provide an integrative foundation for the study of brain, body, and mind. This understanding then allows us to chart a course away from the fallow territory that psychology currently occupies. The integration of the concept of the developmental system with the relational worldview brings forth the importance of considering “co-acting, co-developing processes functioning according to the reciprocal causality entailed by complex positive and negative feedback loops” (Overton and Lerner, 2012, p. 375). As such, the framework of RDS has been offered as an integrative paradigm in which living organisms are understood as dynamic, adaptive, non-linear, self-organizing and self-regulating systems (Lerner, 2006; Overton, 2013). From this perspective, the notion of a system provides a formal explanation, with the directional features of adaptation and self-organization constituting a final pattern explanation (Overton, 2010). RDS recognizes the dynamic complexity of developmental processes and further exposes the inadequacy of split approaches that emphasize simple interaction and the elevation of one level of analysis over another.

In terms of applications of the relational framework, it is important to recognize that RDS is a “mid-range” metatheory that provides a set of core concepts that can inform more specific theories and guide empirical investigation (Overton, 2013). Compatible approaches are those that reject split, mechanistic, or reductionist tendencies and instead put an emphasis on understanding the ontogeny of the individual in the context of the developmental system. One practical example of how this emphasis is realized comes from the family of empirical methods known as person-centered approaches, which in contrast to variable-centered analyses, focus on intraindividual variation rather than on group means (Nesselroade and Molenaar, 2010; von Eye et al., in press).

Finally, if we consider how developmental processes can illuminate the relational ties between different levels, various fundamental questions arise. How can novel structures arise that are different from the sum of their parts? How can activity at one level of explanation account for change at a qualitatively different level? How can the result of “doing more of the same” not simply be “more of the same”? From a much broader perspective, similar puzzles are at the center of the fundamental philosophical problems of intentionality, consciousness, free will, and agency. The underlying question running through these problems involves the problem of relating a level of system or meaning to a level of processes. The conventional approach of isolating or splitting off one of these levels leads directly to the brain-mind or mind-body problems, which are irresolvable when viewed through the traditional lens of analytic philosophy and an associated Cartesian-Split-Mechanistic framework. In moving toward a more embodied framework, the integration provided by relational developmental systems offers a transformation that is based on the fundamental premise that levels of meaning and processes should not be set against each other, but must be viewed as an indissociable complementarity (Overton, 2006, 2010, 2013, in press).

### Conflict of interest statement

The author declares that the research was conducted in the absence of any commercial or financial relationships that could be construed as a potential conflict of interest.
